# Governing stem cell therapy in India: regulatory vacuum or jurisdictional ambiguity?

**DOI:** 10.1080/14636778.2014.970269

**Published:** 2014-10-29

**Authors:** Shashank S. Tiwari, Sujatha Raman

**Affiliations:** ^a^ Institute for Science and Society (ISS), School of Sociology and Social Policy, University of Nottingham, Nottingham, UK

**Keywords:** stem cell therapy, India, STS and biomedical governance

## Abstract

Stem cell treatments are being offered in Indian clinics although preclinical evidence of their efficacy and safety is lacking. This is attributed to a governance vacuum created by the lack of legally binding research guidelines. By contrast, this paper highlights jurisdictional ambiguities arising from trying to regulate stem cell therapy under the auspices of research guidelines when treatments are offered in a private market disconnected from clinical trials. While statutory laws have been strengthened in 2014, prospects for their implementation remain weak, given embedded challenges of putting healthcare laws and professional codes into practice. Finally, attending to the capacities of consumer law and civil society activism to remedy the problem of unregulated treatments, the paper finds that the very definition of a governance vacuum needs to be reframed to clarify whose rights to health care are threatened by the proliferation of commercial treatments and individualized negligence-based remedies for grievances.

## Introduction

India is a key player in the stem cell sector with significant government investment in this area and research activities including the creation of new embryonic cell lines and publication of scientific papers (Inamdar *et al*. 2009; Sharma 2009; Tiwari and Desai 2011). While these efforts have been commended nationally and in the international community (e.g. Lander *et al*. 2008), significant concerns began to emerge from the mid-2000s over unproven stem cell treatments being offered in clinics with apparently little by way of regulatory oversight (Jayaraman 2005). In 2014, it appears that the Indian government has responded to these concerns by announcing legal changes that would, in theory, outlaw stem cell therapies given the absence of clinical trial evidence of their safety and efficacy (CDSCO 2014). In this paper, we aim to make sense of why stem cell activities have been historically difficult to govern and the implications for the implementation of recent regulatory developments (CDSCO 2014; ICMR-DBT 2013).

Various Indian clinics have been accused of making false claims about the efficacy of a wide range of stem cell treatments and, in some cases, offering fake declarations of approval from governing bodies (Pandya 2008; Sipp 2009). The reported use of embryonic stem cell therapies by Nutech Mediworld in New Delhi attracted widespread condemnation (Basu 2005; Cohen and Cohen 2010; Khullar 2009; Padma 2006; Ramesh 2005; Srinivasan 2006). Other claims related to clinical use of adult stem cells have also been controversial; for example, Lifeline Hospital in Chennai claimed that an injection of stem cells can help “improve nerve function” following spinal cord injury, although there is no clinical evidence that this is yet possible (Pandya 2008). A rise in so-called stem cell tourism with patients from the West traveling to India to be treated has been particular cause for concern in international commentary (Cohen and Cohen 2010). Such uses of stem cells are taken to be “experimental”, in that the regimes in question are yet to be proven treatments established as such through a recognized framework of clinical trials. In the absence of evidence that international and national guidelines on stem cell treatments were being followed and the apparent inability of Indian regulatory agencies to rectify the situation, scholars and practitioners have argued that stem cell development in India operated in a “governance vacuum” (Salter 2008).

Concerns about regulatory shortcomings around stem cell activities are not unique to India. For example, while some commentators characterize the problem in India as a departure from “internationally accepted” standards for biomedicine (e.g. Cohen and Cohen 2010), others (e.g. Qiu 2009; Sipp 2009) highlight examples of unproven/untested stem cell treatments being advertised and offered in a range of countries including the USA, the birthplace of codified discourse on medical ethics and bioethics. Some of this links to the rise of Internet advertising of new treatments, the proliferation of cross-border networks and the challenges of governance in such a deterritorialized context. The emergence of unproven stem cell therapies in other countries including China, Hungary, Russia Thailand and some parts of Europe (Bianco *et al*. 2013) and an associated rise in international “stem cell tourism” have also been noted (Cohen and Cohen 2010; Qiu 2009; Sipp 2009). While we focus in our paper on India, this case should be understood within the wider global political economy of stem cell activity.

The Indian stem cell sector sparked significant social science interest from the mid-2000s onwards with several studies concluding that the governance vacuum in India was a result of the lack of *statutory* regulation of stem cell activities (Cohen and Cohen 2010; Glasner 2009; Patra and Sleeboom-Faulkner 2009, 2010; Salter 2008; Salter *et al*. 2007; Sleeboom-Faulkner and Patra 2011). In 2007, the Indian Council of Medical Research (ICMR) and the Department of Biotechnology (DBT) jointly issued a set of *Guidelines for Stem Cell Research and Therapy* (ICMR-DBT 2007). This 76-page document specified general ethical principles for research and processes for formal committee approval of stem cell activities and for their periodic review/monitoring. In terms of procedures and underlying norms, the content was in line with mainstream bioethics. The Guidelines stipulated that clinical use of stem cells was not permitted and that any use of stem cells in clinical contexts (with the exception of already standardized uses of bone marrow transplantation and epithelial therapies for corneal disorders) must be part of a clinical trial conducted after approval by a committee set up to oversee stem cell activity the Institutional Committee for Stem Cell Research and Therapy (IC-SCRT), the relevant research ethics committee and the Drug Controller General of India (DCGI) who sits within the Central Drugs Standard Control Organization (CDSCO). However, since these guidelines lacked statutory backing, many scholars concluded that the way forward would be to give them legislative weight. Yet, the picture emerging from the social science literature on the broader complexities of India's stem cell sector has not been matched by a similar approach to the complexity of law and regulation.

For example, most authors acknowledge the ethical and political conundrum of high-tech stem cell activities taking place in a context of extreme inequalities in income and in access to basic health care (Bharadwaj and Glasner 2009; Glasner 2009; Patra and Sleeboom-Faulkner 2009, 2010; Salter 2008; Sleeboom-Faulkner and Patra 2011). Bharadwaj (2012, 2014) also reminds us that the meanings of stem cells are culturally specific, thus implicitly opening up the assumptions that underlie the critique of unproven therapies being offered in India. Patra and Sleeboom-Faulkner (2010) consider how Indian stem cell researchers interpret the ICMR-DBT guidelines on the ground, providing the first clue that the manner of implementation is at least as important as the making of new laws. However, social scientists have yet to examine the governance of stem cell activities in India asking basic questions such as the following: How has the governance problem around stem cells been framed in India? What are the possible pathways for governing stem cell activity, including, but not restricted to, statutory guidelines? What does the effort to debate and govern stem cell therapy conceal as well as reveal about India's engagement with biomedicine?

In this paper, we address these questions by drawing on approaches in STS and socio-legal studies that help us conceptualize the ambiguity of governing “therapeutic contexts demarcated as experimental sites” (Bharadwaj 2014, 85). In the process, we highlight an unspoken tension in social science work on the Indian stem cell sector with some investigations taking the “problem” of a vacuum in governance as a given (Glasner 2009; Patra and Sleeboom-Faulkner 2009, 2010; Salter 2008; Sleeboom-Faulkner and Patra 2011) and others implicitly challenging the framing of “maverick” science *as* a problem to be governed (e.g. Bharadwaj 2014; Bharadwaj and Glasner 2009). Yet, this tension can be a productive one by keeping law as a mode of governance in sight while prompting new questions about it.

Our work is grounded in STS approaches to science, law and governance (Cloatre and Pickersgill 2015; Jasanoff 2005, 2011b), key insights from which are summarized in the first section. We then examine the significance of how the “object of governance” is constituted in the Indian stem cell case, paying specific attention to boundaries of jurisdiction between different regulatory agencies in the domains of biomedicine, biomedical research and stem cells. Glasner (2009) argues that stem cell therapy in India exists in a liminal space between accepted global standards and local, cultural ones. But liminality applies not just to the global/local, but to the space that straddles research and clinical practice as we explore in this paper.

Second, the focus on a statutory gap as the underlying cause of legitimacy problems seems to be based on the assumption that laws, once enacted, automatically coerce people to behave in the ways intended by their designers. Yet, scholars in socio-legal studies have long highlighted the limits of a purely “top-down” approach to understanding the nature and abilities of state intervention and of law itself (e.g. Kagan, Gunningham, and Thornton 2003; May 2005). Issues of meaning, discretion and judgment remain important in the domain of more formal and codified laws. In democratic societies, when law “works” – or when it is seen to work – it is through a process that unfolds and becomes enacted *through* society rather than being imposed *on* it. This in turn also means that non-statutory guidelines do not necessarily have to produce disorder or ethical transgressions as social or professional norms sometimes produce law-like behavior (Jasanoff 2011b). The failure of India's stem cell guidelines therefore needs to be *explained* rather than assumed to be the natural outcome of their non-statutory status. Multiple approaches to governing health care in India are now emerging beyond those represented by statutory laws alone (Peters and Muraleedharan 2008) and we examine their significance for the implementation of recent stem cell laws. In this context, we ask what efforts to debate and govern stem cell activity *exclude* as well as include in shaping the problem at stake.

## Law, society and the making of governance

The struggle to regulate the objects and practices of the biosciences and technologies has been extensively investigated in STS (e.g. see chapters in Cloatre and Pickersgill 2015 and in Jasanoff 2005, 2011a; Kim 2013; Patra and Sleeboom-Faulkner 2010; Raman and Tutton 2010; Sleeboom-Faulkner and Patra, 2008; Sunder Rajan 2007). At stake for law and law-making systems is the question of whether biotechnologies constitute entirely novel domains of intervention – therefore needing new legal perspectives and instruments for their governance – or if they should be seen as incremental extensions of current activities – in which case, existing systems might be adequate. Jasanoff (2005) shows that different national systems have engaged with these questions differently in ways shaped by their political cultures. The comparative approach adopted in this work stimulates some fundamental questions about the making of biotechnology governance that are relevant even where we might focus on particular national systems as in this paper.

First, how do particular activities come to be seen as risky or needing regulation while others do not – and vice versa? A central problem for biotechnology governance in all countries is the uneasy tension between the imperative to promote new technologies and the imperative to regulate them. The establishment and perceived legitimacy of a regulatory apparatus for biomedical research has helped governing bodies in Britain and the USA to manage this tension in different sectors, though the extent to which these attempts are successful varies over time and sector (for example, questions periodically emerge over the lack of regulation of US stem cell activity in the private sector or over the activities overseen by the Human Fertilisation and Embryology Authority in Britain). In historical terms, the tension has been transformed into a productive one for scientists with regulation seen as a way of managing reputational risk (Dixon-Woods and Ashcroft 2008), and in that respect, enabling rather than only constraining research. Comparing the US response to crop biotechnology with that of Britain and Germany, Jasanoff (2005) argues that the early lack of concern in the USA was in keeping with the state's reliance on science as a mode of handling controversy. In the 1980s, molecular biologists framed the technology as an extension of established techniques, thus helping to keep crop biotechnology politically invisible; a similar approach in Britain was initially successful, but the bovine spongiform encephalopathy crisis altered the status of science advice and helped to “open up” the regulation of biotechnology. Scandals can help galvanize a case for regulation, though the outcome depends on how such a case is framed.

Second, when new objects of governance are made visible as needing intervention, how are they constituted and ordered? For example, both human and agricultural biotechnologies have been constituted as a series of *products* in the USA, thus allowing them to be regulated by existing frameworks of contract law for market transactions with any grievances handled through the courts (Jasanoff 2011b). By contrast, countries like Britain handled technologies such as those relating to surrogacy through family law. Different ways of constituting the object lent themselves to different spheres of jurisdiction, the political and cultural legitimacy of which allowed governments to manage controversies around new technologies. Research on law-making therefore highlights the value of looking beyond the statutory status of guidelines to ask how legal or regulatory questions are framed in the first place and how this matters for jurisdictional boundaries.

Third, it is not enough to look at how formal laws and policies come into existence since professional and political norms of practice and judgment may acquire law-like qualities despite never having been formally articulated as such (Jasanoff 2011b). This connects with work in political science on ways of thinking about governance (Pierre and Peters 2000), as well as with STS notions of “scientific governance” (Irwin 2008) where governance or the creation of order is understood in terms of the interaction between different mechanisms rather than necessarily centered on the state. These might include activities of the professions, industry and civil society as well as government. For example, civil society groups might put pressure on the professions, industries or the state in order to hold them to account in terms of their role in governing. This can also work across borders in a “glocalization of law” (Randeria 2003) where civil society groups draw on international standards to contest policies introduced by particular state agencies (see also London and Schneider 2012). Writing on the Bhopal disaster, Jasanoff (1988) argues that the effectiveness of right-to-know laws depends in part on acknowledgements of the institutional duty to disclose information and the rights of citizens to participate in choices about technology. In the case of medical treatment, these choices tend to become individualized – however, activists and civil society groups could play a crucial role by making relevant information more public.

Fourth and related to the above, a key question is what happens to statutory laws once they come into existence. Asking this question also opens up the possibility of interactions between (different levels within) the state, professions or other communities, industries and civil society in the process of implementing formal laws and policies. For laws to have social meaning “they must become embedded in people's imaginations and understandings, and worked out in their practical dealings with one another” (Jasanoff 2011b, 15). The flip side is that some laws may exist only in the rulebooks rather than in practice, producing an “implementation gap.” Others may be re-interpreted, reshaped and given new meanings through the course of being implemented in various settings. Scholars in law and society have examined factors shaping compliance, non-compliance and, in some cases, “over-compliance” with the law by different actors in various domains (Kagan, Gunningham, and Thornton 2003; May 2005). These studies pinpoint a variety of potential factors influencing behavior: fear of sanctions; visibility and frequency of inspections/monitoring; level of trust in public institutions; fear of threats to reputational risk; and normative beliefs about “doing the right thing.” In exploring the relevance of these issues of law-in-practice to Indian stem cell activities, we draw on related research on health care law and ethics in India (Madhiwalla 2011; Peters and Muraleedharan 2008; Thatte, Kulkarni-Munshi, and Kalekar 2009). For example, a review of Indian policies around participant injury in clinical research found that researchers were largely unaware of their responsibilities (Thatte, Kulkarni-Munshi, and Kalekar 2009).

Finally, bringing a civil society perspective into the study of law opens up the possibility of fundamentally rethinking the terms on which the regulatory problem at stake has been framed in the first place. Investigating stem cell governance in South Korea, Kim (2013) argues that the relevant question is much more than about the way to deal with research misconduct, an issue on which much commentary has centered in the aftermath of the Hwang Woo Suk scandal. Civil society activists have instead argued for stricter controls in order to deal with potential threats to the public interest from a dominant capitalist-developmental drive toward biotechnology. Likewise, Sunder Rajan (2007) reframes the conventional focus on informed consent in Indian clinical trials with the socioeconomic one of who benefits from such research. As Cloatre and Pickersgill (2015) argue, legal engagements with science represent particular visions of how we should live and exclude alternative futures. Research on stem cell governance needs to acknowledge the inequalities entailed in the politics of life (Raman and Tutton 2010) which, in this case, means being sensitive to why a governance vacuum matters and to whom.

## Methods

To understand the making and interpretation of law-in-practice, it is essential to consider how stakeholders “on the ground” perceive the key issues – in this case, difficulties around stem cell governance and prospects for their remediation. Hence, a qualitative study of documents in different media (news and opinion, scientific literature, policy reports), and interviews with key stakeholders was undertaken.

Semi-structured interviews were conducted after ethical approval from the University of Nottingham during June 2010–January 2011, and again during September–October 2011, in various cities in India including New Delhi, Mumbai, Pune, Chennai, Bangalore, Hyderabad, Tirupati, Kolkata and Chandigarh where most of the research and clinical activities in stem cells are being carried out. Locations were identified on the basis of a mapping exercise in 2010 using documents available on the Internet. 27 interviews (5 scientists, 11 clinicians, 7 firms' representatives and four policy-makers) were conducted by the first author, lasting between 45 minutes and an hour (with one exception, where the interview finished in 15 minutes). The majority of the interviews were recorded with the informants' permission, and transcribed. However, in three cases, informants were not comfortable with the prospect of being recorded; hence, notes were taken and subsequently written up.

Documents included news-items on stem cell activities published between 2001 and 2012 in leading newspapers available on the Internet (*The Times of India*, *The Hindu*, *The Indian Express*), science magazines (*BioSpectrum India*), official documents related to stem cell research and medical governance published by government bodies, and articles published in international journals on stem cells in India (e.g. *Nature*, *Science*). There is a lively debate in Indian newspapers and journals (especially the *Indian Journal of Medical Ethics*) on the state of medical ethics in the country. These articles also provided key insights into how parts of the medical profession in India perceive the issues that are being discussed elsewhere in the international media and journal literature. Finally, in updating the research for this paper, current news-items were included to take on board recent developments in stem cell regulation and developments reported in the Indian media around medical negligence.

## Constituting stem cell research as the object of governance

In addition to the non-statutory status of the 2007 ICMR-DBT guidelines, scholars have identified a fragmentation of regulatory authority as a problem for stem cell governance (Patra and Sleeboom-Faulkner 2009; Salter *et al*. 2007). The ICMR is part of the Ministry of Health and Family Welfare, while the DBT is in the Ministry of Science and Technology. Yet, such a structure does not necessarily have to fail as it might represent an effective way of combining forces in complex situations calling for multiple sources of expertise. Following Jasanoff's (2011b) injunction to consider how biotechnology is ordered, we ask how stem cell therapy has been constituted as a regulatory object. Framing the question this way sheds light on the terms in which the “problem” to be regulated is made visible and jurisdictional boundaries drawn, which, in turn, allows us to consider if these boundaries might be defined differently.

In the early days, the Indian government was keen to promote Dr Shroff's work with the then Health Secretary quoted in 2005 as saying that “sometimes, scientific knowledge cannot wait for bureaucratic apparatus” (Mudur 2005). However, fears about reputational risk (Dixon-Woods and Ashcroft 2008) began to emerge and stimulated a regulatory response. Many Indian scientists and clinicians expressed concerns to journalists about unwarranted claims made by Dr Shroff and others (e.g. Lifeline Hospital, Chennai; All India Institute of Medical Sciences, New Delhi) regarding successful treatments based on stem cells (see Mudur 2005). A few took to journals in science and in medical ethics to criticize these claims and the lack of an effective response from government (e.g. Jayaraman 2005; Padma 2006; Pandya 2008). Some of these criticisms came from stem cell scientists concerned that those making unverifiable and overstated claims about stem cells posed a threat to the reputation of others working according to established norms of research. Many urged the ICMR to take a firmer stance and “mandate” medical ethics (Mudur 2005).

Given their keenness to secure investor confidence in biomedical research (Sunder Rajan 2007), it is not surprising that the Indian government responded quickly to these developments with the 2007 ICMR-DBT guidelines that were modeled on established liberal-bioethical frameworks. However, critics argued that these needed to have legislative force (e.g. Pandya 2008), a point that was also evident during interviews conducted with key players. One scientist working in a government-funded research laboratory observed with reference to the ICMR-DBT guidelines that

Now it has to pass through parliament as a rule and once it is made as a rule then probably those malpractices will be stopped, otherwise it will not stop. (Scientist 1)

He also argued that those who violated the guidelines should be punished. A private medical practitioner, who himself offers experimental stem cell therapy for muscular dystrophy, lamented that everyone was free to offer stem cell therapy:

[The] government of India has policies but I think they are sluggish … the legislation in India does not have any teeth on the stem cell therapy providers … currently there is no law for that; just a guideline if you violated nobody is bothered. (Clinician 1)

This seems to confirm the point made in the social science literature on Indian stem cell activities (Patra and Sleeboom-Faulkner 2010; Salter 2008) that guidelines cannot compel action in the way that laws ostensibly can. However, while the development of guidelines as a response to controversy was seemingly straightforward, the question of jurisdictional authority over their implementation has been more complicated. What was missed in this debate was the fact that neither the ICMR nor the DBT has a *legislative* remit over medical research. Interviewees in government and industry pointed out that the ICMR funds research and provides advice, while the DBT is an agency for funding (rather than regulating) preclinical and clinical R&D. Also, the DBT has no remit over activities taking place outside government-funded R&D programs (Policy-maker 2). Its stem cell task force and committees oversee the DBT's own research activities, but these do not cover clinical trials.

The DCGI which is frequently characterized as the “Indian FDA” already had a mandate to regulate clinical trials and would have been the obvious candidate to extend its remit to stem cells. Only the DCGI has the authority to regulate their activities, an industry representative was quick to point out (Firms representative 2). However, the DCGI had no experts of its own who were able to evaluate stem cell proposals, according to a policy-maker. Also, in these early days, it appeared that the DCGI was uncertain about the reach of its powers which may be due to the fact that it is only nominally similar to the FDA with a remit primarily related to drug approvals (Sunder Rajan 2007). “Our FDA is not that strong” noted a policy-maker (Policy-maker 2). Clinician 1 quoted above added:

Even if you go to the Drug Controller of India he says, what can I do … when I don't have powers to crush you, even if you don't follow the guidelines why should I bother you? (Clinician 1)

This suggests a fundamental jurisdictional ambiguity with even the relevant agency unsure of what falls under its regulatory scope. The following quote from a policy-maker explains more clearly the reasons for this ambiguity. Referring to the wider landscape of medical law, this policy-maker suggested:

whatever they (i.e. doctors) do in the name of research they should follow the ICMR guidelines *but the problem with the stem cell therapy is that those who are offering therapy don't consider it as research … OK … they think that it is therapeutic* … any doctor has the right to give treatment … so they are doing their practice … it (does) not come under research … so there is no need for any permission and there is no need to follow any guidelines … you really can't punish them. (Policy-maker 3, emphasis added)

Here, we begin to see that the jurisdictional difficulties around identifying who has authority to regulate stem cell “research” have arisen partly from the ambiguous boundary between research and therapy. For those offering stem cell therapy, guidelines pertaining to “research” do not appear to have meaning since they seem themselves as treating patients rather than using them as research subjects for publishable studies (see also Bharadwaj 2014). Indeed, comparing the 2007 guidelines with the draft revisions published in 2012 (ICMR-DBT 2012) and subsequently finalized in 2013, the most dramatic change relates to the very title. In 2007, the document was labeled *Guidelines for Stem Cell Research **and Therapy*** (emphasis added). By 2012/2013, this now reads *Guidelines for Stem Cell Research*. The writers of the Foreword to the 2013 document draw attention to the change, explaining that this was done to avoid confusion over the fact that stem cell therapy is not allowed in the first place, hence there can be no guidelines to govern it! The 2013 guidelines reiterate the point that any clinical use of stem cells must be part of an authorized clinical trial, a point that was already present in the 2007 version, but did not have meaning for those carrying out the offending activity.

To summarize, constituting the regulatory object as “research” enabled the ICMR to bring its expertise in stem cells and bioethics to bear on the problem. However, since the ICMR can only provide advice, the DCGI's statutory powers were highlighted as the answer to the problem of ICMR's guidelines being ignored in practice. However, insofar as unproven therapy was being provided in clinical settings *outside* recognized clinical trials, bringing this activity under DCGI's remit proved challenging in the first instance.

This marks a key difference from the scandals that stimulated the codification of medical research regulation in the West (Dixon-Woods and Ashcroft 2008). Where those controversies were sparked by ethical questions raised by clinical trials with clinicians appearing to pursue research goals rather than the needs of (some) patients, the issue in the Indian context is not about research per se (defined as that occurring in the context of clinical trials) but a *market* for experimental treatments. Strikingly, none of the clinicians interviewed as part of this study spoke about conducting clinical trials or even “research,”; rather, their focus was on stem cells as a therapeutic intervention offered in most cases for a fee.

### Reconstituting stem cell governance

So far, we have highlighted the jurisdictional ambiguities that challenge one-dimensional accounts of a governance vacuum in stem cell research. Yet, jurisdictional boundaries and the objects of regulation can be open to re-constitution as was evident at the time of fieldwork and confirmed by recent developments in stem cell governance. In light of the persistent controversy over stem cell therapies, the story of what the “Indian FDA” can or cannot do was slowly being opened up to alternative interpretations in interviews conducted in 2010–2011. For example, one policy-maker observed:

Initially, DCGI thought that stem cell does not fall as a biological entity so they say that it does not come under their purview so they started forwarding … all these applications to ICMR. But later on they realised that it is part of schedule Y, it comes under biological cell or vaccine or recombinant. (Policy-maker 1)

The point here is that as clinicians begin to offer marketable stem cell products, they were opening themselves up to scrutiny by the DCGI under its existing remit which (unlike the DBT) covers both public *and* private activities.

In 2012, the DCGI constituted a special division for stem cells in response to criticisms that it does not have any internal evaluation mechanism (*BioSpectrum India*, 30 April 2012). In practice, DCGI's reliance on the ICMR is set to continue as ICMR's Director-General is also the Chairman of the new division, though as one interviewee noted, both agencies are part of the same Ministry and this co-working needs not be construed as a problem (Policy-maker 2). The government of India also set up a long-awaited National Apex Committee for Stem Cell Research and Therapy (NAC-SCRT) to oversee and monitor activities in this field. In 2014, in addition to the publication of revised guidelines mentioned above from the ICMR-DBT, the DCGI announced that it would modify the Drugs and Cosmetics Act to treat “stem cells and cell-based products” as new drugs (CDSCO 2014). With this announcement, it appears that the regulatory vacuum in the Indian stem cell sector is finally being addressed by statutory law. Yet, if the meaning of law is determined in practice, legal amendments are insufficient in themselves to draw a line under the challenges of stem cell governance. While the DCGI has addressed the ambiguity over who has legal jurisdiction over stem cell uses, we still need to know how medical law works on the ground in order to make sense of the practical implications of DCGI's efforts. In the next section, we explore ways in which clinical medicine is regulated in India and their prospects for contributing to the governance of stem cell therapy.

## Enacting stem cell governance through regulation of clinical practice?

For punishing you should have a strong mechanism so that you know … somebody has to complain … the complaint has to be seen by somebody and then you can take it to human rights or anything for the punishment … otherwise you can't do anything. (Policy-maker 3)

Jurisdictional ambiguities over the governance of stem cell therapy seem to have finally been resolved with the ICMR-DBT revising their guidelines and the DCGI extending their statutory remit to stem cells in 2013–2014. Yet, even well-ordered statutory laws require mechanisms for enforcement. In the above quote, our interviewee implicitly raises the possibility of potential violations altogether going unnoticed. In this section, we consider two possible routes by which recent stem cell laws may – or may not – be enacted in practice, first, through professional self-regulation and second, through the broader edifice of statutory law governing clinical practice. We examine the difference, if any, that law makes through its interaction with the medical profession and the courts.

International and national guidelines stipulate that clinical uses of stem cells must be as part of a clinical trial for research conducted under established regulatory protocols. If, as we have argued, stem cell therapy escaped the regulatory net due to its location in a health care market rather than research per se, one option might be self-regulation through ethical codes of conduct that cover clinical practice. Indeed, if medical ethics predates the development of ethics for medical research, this seems an obvious response. Specific norms can emerge and acquire law-like qualities (Jasanoff 2011b) through the process of medical education and subsequent membership in a professional community. The state plays a role here by validating the profession and providing an overarching structure within which medical providers govern themselves. If this type of normative behavior has not emerged around stem cell therapy, we need to consider why.

Sanctioned by the Indian Medical Council Act of 1956, the Medical Council of India (MCI) is the primary regulatory body for maintaining uniform standards of medical education and certifying medical qualifications. Medical practitioners register through state-level councils overseen by the MCI. In 2002, the MCI introduced the *Indian Medical Council (Professional Conduct, Etiquette and Ethics) Regulations, 2002* to cover codes of conduct for practitioners, which again operate through state councils. However, violations of the code have been noted and the code itself challenged as impractical in a highly market-driven health care sector. For instance, some clinicians and corporate hospitals advertise their medical services through media interviews or hoardings at public places although the medical code considers advertising to be unethical (Balasubramanian 2008). Overall, the MCI, at both central and state levels, is perceived to be ineffective in monitoring codes of conduct with critics charging that “they have not bothered to exercise the powers given to check unethical medical practice” (Pandya 2007, 2). The MCI and state medical councils have also been plagued with corruption charges over the years (Pandya 2007; *The Times of India*, 24 April 2010).

In addition to state-sanctioned councils, the Indian Medical Association (IMA) is the main professional body for doctors. Its website highlights that “[IMA] looks after the interest of doctors as well as the well-being of the community at large.”^1^ However, in a stinging critique, one doctor charges the IMA with behaving “as an interest group pushing the special interests of doctors instead of society as a whole” and altogether failing to contribute to policy on improving health indices in India (Thomas 2011, 2). Dr Thomas also takes the MCI to task for failing to provide leadership on ethics education for doctors (Thomas 2011).

What, though, are the reasons for the failure of ethical codes to be translated into practice? Madhiwalla (2011, 3) argues that the medical profession in India has not traditionally faced the type of public scrutiny that medicine received in the West owing to its origins as a sector built “by both the colonial and the independent Indian state as the vehicle of modernity and welfare.” An interest in bioethics emerged in the 1980s from controversy over the role of medicine in the 1984 Bhopal disaster and earlier, in sterilization programs introduced during the 1975 Emergency. However, it remained a “niche” interest with few roots in professional education. As India emerged as a key provider in a newly globalized health industry, formalized procedures and frameworks for ethics have been eagerly embraced (Madhiwalla 2011) and actively promoted by contract research organizations (CROs) seeking to secure investor confidence in the country as a site for clinical trials (Sunder Rajan 2007). But this has happened almost too quickly “without the churning, debating and refining of ideas and concepts, application to practice and critique of that practice, the breaking and formation of public opinion, the coming together and parting ways of different groups … ” (Madhiwalla 2011, 3, italics added) that is central to the process by which law and law-like guidelines acquire social meaning (Jasanoff 2011b).

Others emphasize the need for guidelines to be backed up by threat of sanctions. Commenting on the lack of efficacy of the MCI's code of conduct, one doctor said, “it is important to have ethical guidelines. But the profession should enforce them. We need to develop mechanisms so that a variety of transgressions are regulated and penalised” (Dr K. Reddy quoted in Jain 2010). Once again, it seems there is no escaping the hopes pinned on statutory law. However, the question is how violations of laws or professional codes become visible in the first place. Who notices if something goes wrong? We turn to this question below.

At present, medical ethics violations are dealt with indirectly under various sections of the Indian Penal Code which defines criminal acts and related punishments (Dhar 2010). Section 304-A of the Code deals with complaints against medical practitioners for alleged medical negligence (Nayak 2004) which includes violations of medical ethics (Dhar 2010). The civil law of torts is considered to be among the most significant for governing medical malpractice as it has been successfully applied in many cases (Peters and Muraleedharan 2008). It applies to all health professionals, whether in the public or the private sector. This law also covers circumstances when a clinician treats a patient without informed consent (Nandimath 2009). The Indian Contract Act of 1872 provides legal protection to agreements between the parties, but has hardly been used for health issues in India (Peters and Muraleedharan 2008).

Taken together, these legal avenues appear to offer some statutory weight for governing medical practices including, unproven stem cell treatments. So, if such treatments were offered despite recent legal amendments, these cases could, in theory, be pursued by underpinning legislation such as the Indian Penal Code. However, the social meaning (Jasanoff 2011b) of any of these laws as they have been applied in the medical sector is problematic, given the way in which they have been tended to be interpreted in the courts and entrenched delays in completing court cases. According to one Indian Supreme Court order, the opinion of an expert or panel of doctors is necessary to begin a case (Kamath 2010). It is also alleged that courts have tended to favor medical providers in their rulings (Peters and Muraleedharan 2008). For example, in one hearing, the Supreme Court stated that

 … it is the bounden duty of civil society to ensure that the medical professionals are not unnecessarily harassed by complainants who use the criminal process as a tool for pressurising the medical professionals and hospitals for extracting uncalled for compensation. It would not be conducive to the efficiency of the medical profession, if a doctor is to administer medicine with a halter around his neck. (quoted in Menon 2010, 96)

Until 2010, more than 30 million cases were pending in various courts of India and one source estimates that it will take 320 years to clear all cases (*The Times of India*, 6 March 2010). Second, some people who might be able to secure a way of financing stem cell treatments in a context of desperation, may not be able to extend these resources to fight for several years for justice should they have grievances. A recent high-profile case of alleged medical negligence which resulted in a hefty payout to the plaintiff in question is perhaps the exception that proves the rule. In October 2013, the Supreme Court awarded Kunal Saha, a US resident the highest ever compensation (nearly $1 million) in a medical negligence case in India following allegations against a hospital in Kolkata which treated his (now deceased) wife (BBC, 24 October 2013). However, Saha originally launched his case in 1998 and was able to sustain it through various legal twists and turns. Clearly, this would be out of the question for most people.

In light of such challenges, Peters and Muraleedharan (2008) suggest that focusing on enforcement of legal mechanisms is insufficient since “the limited ability to enforce civil and criminal laws in India is well known” (Peters and Muraleedharan 2008, 2137). For instance, the implementation of the 1994 Preconception and Prenatal Diagnostic Techniques (PCPNDT) Act was made possible only after the intervention of the Supreme Court in 2000 (Kurup 2011). Similar points about the limitations of enforcement have been made in relation to other domains such as the Biological Diversity Act of 2002 (Bhutani and Kohli 2012). Peters and Muraleedharan (2008) therefore call for approaches focusing on the capacity of consumers to raise complaints through alternative forums. This then opens up the possibility of making sense of stem cell governance through a wider perspective offered by investigations of the relationship between law, medicine and civil society, a question to which we now turn.

## Stem cell governance through civil society

If neither statutory laws nor professional self-regulation is sufficient for governance, we need to ask how law may be supported or given meaning through its embedding in civil society. Peters and Muraleedharan's (2008) approach to this involves looking for civil society-centered mechanisms for improving the efficacy of law-in-practice. However, STS and socio-legal approaches (Cloatre and Pickersgill 2015; Kim 2013) provoke a more radical re-opening of the very question around which the notion of a “governance vacuum” in the Indian stem cell sector has emerged. We consider each of these in turn.

One way to deal with the limits of the court system is to develop alternative mechanisms of enforcing medical laws. Here, the Consumer Protection Act (CPA) 1986 is potentially relevant as it is meant to protect the interests of consumers from poor-quality products/services and provide quicker responses to grievances by circumventing the delays of court cases (Peters and Muraleedharan 2008). Cases are brought to Consumer Forums which do not require court fees. Medical services were included in this Act in the year 1995 after a Supreme Court ruling that “patients aggrieved by deficiencies in medical services rendered for payment can claim damages under the act” (Mudur 1995, 1385). However, complainants still do have costs and delays beyond the stipulated three-month limit remain a problem. Unsurprisingly, most health cases tend to be brought by wealthier and educated families (Peters and Muraleedharan 2008).

Also, the CPA covers only private clinicians who offer paid services. As with the Indian Penal Code, the CPA requires expert advice from other doctors for a case of alleged malpractice or negligence to be brought (Joshi 2011). This Act also does not cover clinicians working in public hospitals. In theory, this gap should not be relevant to the case of stem cell treatments which are primarily offered in private practice for a fee while services in government-run public hospitals are, for the most part, free. However, the link between private and public health care is more blurred in practice with public-sector doctors doing private practice and sometimes referring their patients to private facilities for certain services. This may also account for the fact that the private sector accounts for 80% of health care services in India (Peters and Muraleedharan 2008), despite high levels of poverty.

If public-sector medicine does become relevant to the governance of stem cell therapy through interfaces with the private, the gap in the CPA could, in principle, be addressed through right-to-know laws (Jasanoff 1988) which allow individuals to access information held by public authorities. India introduced a Right to Information Act in 2005 which has been used over the years by activists to seek information on clinical trials and publicize ethical violations (Paliwal 2011). Mere exposure of violations does not guarantee that perpetrators will be held to account. For example, in one case in Madhya Pradesh, government doctors were found to have made millions of dollars through their role in corrupt clinical trials though this resulted in a mere $100 fine (Yee 2012). Still, it is worth asking if such outcomes might be transformed in future through civil society activism.

Civil society movements have been active in India around a number of science-related domains including the Chipko movement related to environmental protection, action around the Bhopal disaster (Jasanoff 1988) and development priorities, the anti-GM agriculture movement (Scoones 2008) and, more recently, the anti-nuclear power movement (Srikant 2009). Some of these have impacted state decisions; for instance, protests against Bt Brinjal, a genetically modified crop, forced the Indian government to postpone the approval of commercial release of this technology. Indeed, at the time of writing, the Indian government has been sufficiently worried about the impact of environmental and development NGO challenges to the agenda of unfettered economic growth to brand many of these activists as “anti-Indian” (Ranjan 2014).

The role of organized civil society action around biomedicine is similarly becoming stronger; however, the capacity to impact “high-tech” biomedicine is more complex. Bhattacharya, Proctor, and Hodges (2008) claim that the exploitation of poor people during clinical trials and surrogacy or issues related to the trade in organs and human tissues have gone unnoticed, failing to create a mass movement. Controversial HPV vaccine trials did, however, spark organized action and led to the trials being suspended (Sarojini *et al*. 2010). Following protests in February 2011 around clinical trials involving victims of the Bhopal gas disaster (Rajalakshmi 2012), the government of India was compelled to take action against clinicians who were involved, though health activists subsequently criticized the penalty imposed as a mere token (Yee 2012). Such episodes helped to trigger the government effort in 2013 to amend and strengthen the 1940 Drugs and Cosmetics Act with new powers to punish violations (Times of India 2013). So, while health activism does have a history in India (Madhiwalla 2011), it is only now increasing in national visibility. Medical deference remains widespread, however.

Ultimately, the issue that must be confronted to understand the prospects for governing stem cell treatments is the fact that these are offered primarily in a private market, often at high costs. Patra and Sleeboom-Faulkner (2009) report that Indian patients – including many who travel from other parts of the country to urban centers where stem cell treatments are offered – are mostly from the upper-middle and upper economic strata. “Medical tourists” include patients from other developing countries or from the industrialized countries where approved treatments using stem cells are not yet available, but who are likewise able to find ways of paying for them in India. Much of health activism in India occurs, for good reason, around the needs of the most marginalized and oppressed populations, and so, it is unlikely that absence of oversight in expensive commercial health care services would, in itself, be an issue. For example, the Mumbai-based group Sama states on its website that it “considers health a fundamental human right and believes that the provision of quality and affordable health care to every citizen is the responsibility of the state.”^2^ The group focuses on women's health issues in the wider context of socioeconomic realities, and campaigns against the commercialization of health care. From this perspective, it is those questions that follow from the creation of commercial markets for stem cell treatments that are the most pertinent.

First, do such markets adversely affect health services for the poor by diverting investment that might otherwise be spent on basic health care or more recognized treatments (Patra and Sleeboom-Faulkner 2009)? Second, there are concerns that such treatments increase health care costs more widely, affecting middle-class patients who are less affluent. This also affects the viability of medical negligence law such as the Saha case to serve as a mode of governance, given that increasing litigation can drive up insurance costs. Third, should such treatments become incorporated into properly demarcated clinical trials, who might figure as research subjects in this newly regulated domain and what are their chances of receiving benefits? Sunder Rajan (2007) argues that the real violence in Indian subjects serving as “guinea pigs” in clinical trials is a structural one – there is no guarantee that therapies, once developed and approved, would be made generally accessible at an affordable cost. So, the question with which we began this paper and which largely frames the debate on Indian stem cell governance needs to be put in its proper context.

In sum, the ability to enact stem cell laws through civil society mechanisms remains limited, unless patients perceive their rights to have been violated and have the wherewithal to follow through. So far, there is little evidence of this. More significantly, looking at stem cell governance through a civil society lens allows us open up broader questions as Kim (2013) has done with reference to South Korea. A key question for future work might be to explore opportunities for activists to reframe the concern about a “governance vacuum” around unproven therapies to a social justice concern about the very development of stem cell biomedicine in a context of radical social and health care inequalities.

## Conclusion

We began this paper by asking why it has been difficult to govern stem cell treatments offered in India, and the prospects for this vacuum in governance (Salter 2008; Sleeboom-Faulkner and Patra 2008) to be remedied. Many social scientists as well as Indian stem cell scientists have argued that the answer is to create statutory/legal backing for stem cell research guidelines developed by the two major agencies in the sector, the ICMR and the DBT. Drawing from STS and socio-legal approaches, we argued that this diagnosis of a statutory gap was inadequate since the construction of law and the boundaries of regulatory objects need attention as do the ways in which laws and law-like behavior work in practice through social and institutional interactions (Jasanoff 2005, 2011b). Indeed, the statutory gap in Indian stem cell governance was recently addressed with changes announced in 2014 to the DCGI's legal remit and a revised set of guidelines produced by the ICMR-DBT guidelines at the same time. But questions still remain over the capacity to enact law-in-practice and enforce the new laws.

Our work highlighted a key jurisdictional ambiguity around stem cell therapy. The ICMR-DBT guidelines were framed in terms of stem cell research, but research implies the conduct of clinical trials. Until recently, stem cell therapies escaped the regulatory net of the DCGI as they did not take place under the auspices of a trial, sitting primarily in a private market for clinical services. Patients may have taken the risk – or opportunity – of “therapeutic consumption” (Sunder Rajan 2007) without a system of governmental protection backed up by the ability to enforce sanctions. But this indicates that the reputational controversy around Indian stem cell activities did not affect clinical practice even though it damaged the cause of research and eventually led to the recent amendments. The ability to enact these laws in practice depends on their interactions with the medical profession (specifically, mechanisms such as codes of conduct issued by the MCI), the wider edifice of health care governance (specifically, statutory and quasi-statutory options available through the Indian Penal Code or the 1986 CPA) and civil society activism. However, we found that these too are problematic for a number of reasons. The rise in formal frameworks of medical ethics and research ethics has not yet been accompanied by the deliberation and learning from practice that is normally required to give them meaning (Madhiwalla 2011). If legal violations go unnoticed, a de facto “governance vacuum” would still persist. Medical negligence cases may be on the rise in India, but hardly represent a viable option for the majority, not least for the costs they impose on individuals and on the health care system as a whole. Civil society activism around health is becoming more visible, but this is necessarily centered on remedying the serious inequalities of health care access in a country where the commercial sector accounts for 80% of health care services rather than violations in stem cell treatments per se.

In the end, we need to acknowledge that stem cell treatments are primarily offered to those who can afford them – or, who find the means to afford them. This then means asking not only whose rights are potentially being violated by unethical/unregulated treatments, but what the rise of such commercial treatments means for others' rights to health care. It also means attending to the question of who can currently afford to participate in such a market, and who is effectively excluded at the outset from future markets for better regulated/certified forms of stem cell treatment. The vacuum around stem cell activity in India – be it a vacuum in governance or in bioethical behavior – is more problematic than a simple failure to adequately enforce guidelines through statutory or non-statutory means. Rather, the vacuum encompasses multiple inequalities in the politics of life (Raman and Tutton 2010) that shape the governance and delivery of health care which need to be placed center stage in such debates over biomedical research governance.

## Notes

1. 
http://www.ima-india.org/IMA.html.

2. 
http://samawomenshealth.wordpress.com/about/.
